# Bacterial and fungal co-infections with SARS-CoV-2 in solid organ recipients: a retrospective study

**DOI:** 10.1186/s12985-022-01763-9

**Published:** 2022-03-04

**Authors:** Mojtaba Shafiekhani, Zahra Shekari, Arash Boorboor, Zahra Zare, Sara Arabsheybani, Nazanin Azadeh

**Affiliations:** 1 Shiraz Transplant Research Center, Shiraz University of Medical Sciences, Shiraz, Iran; 2 Shiraz Transplant Center, Abu-Ali Sina Hospital, Shiraz University of Medical Sciences, Shiraz, Iran; 3 Department of Clinical Pharmacy, Faculty of Pharmacy, Shiraz University of Medical Sciences, Shiraz, Iran

**Keywords:** Solid organ transplantation, COVID-19, Co-infections

## Abstract

**Background:**

SARS-CoV-2, a novel corona virus, has caused clusters of fatal pneumonia worldwide. Immune compromised patients are among the high risk groups with poor prognosis of the disease. The presence of bacterial or fungal co-infections with SARS-CoV-2 is associated with increased mortality.

**Methods:**

The electronic data of the liver and kidney recipients, hospitalized in COVID-19 intensive care unit in an 8-month period in 2020 were retrospectively assessed. The documented bacterial or fungal infections alongside with outcome and risk factors were recorded and analyzed by binary logistic regression model and multivariate analyses.

**Results:**

Sixty-Six liver and kidney recipients with positive RT-PCR for SARS-CoV-2 were included this study. Twenty one percent of the patients had at least one episode of co-infection during their COVID-19 course. Bacterial and fungal co-infections contributed to a significantly higher mortality. Urine and sputum were the most common sites of pathogen isolation (45.45% and 36.36%; respectively). The majority of infections were caused by vancomycin- resistant *Enterococci* (30%). *Escherichia coli* stood in the next position with 23.3%. Prior hospitalization and high doses of corticosteroids were associated with co-infections (p < 0.001 and p = 0.02; respectively.)

**Conclusions:**

Bacterial and fungal co-infections with COVID-19 are more prevalent in solid organ recipients compared to the general population. Prior hospitalizations and use of broad-spectrum antimicrobial agents lead to emergence of multi-drug resistant pathogens in this susceptible patient population. Early detection and treatment of co-infections as well as antibiotic stewardship is recommended in solid organ recipients.

## Background

Since December 2019, the world has faced a challenging infectious disease called “Coronavirus disease 2019 (COVID-19)”. Since then, the highly contagious disease has caused financial and medical challenges and widespread lockdown throughout the globe [1]. The rapidly spreading disease is caused by the severe acute respiratory syndrome coronavirus 2 (SARS-CoV-2), a novel strain of the coronaviruses [2]. Although generally harmless, coronaviruses have previously gained notoriety for outbreaks of viral cases of pneumonia including severe acute respiratory syndrome (SARS) and Middle East respiratory syndrome (MERS) [3]. Since a few months after the emergence of the new disease, Iran has faced a challenging number of COVID-19 cases and mortality among the middle-eastern countries [4]. Among the patients, solid organ transplant (SOT) recipients are a notable matter of concern. Receiving immunosuppressant poses the SOT patients to higher risk of developing symptomatic COVID-19 disease, compared to the normal population [5]. A French study has reported a COVID-19 incidence of 5% in kidney recipients; while the incidence of the disease was 0.3% in the healthy population [6]. The management of COVID-19 in SOT patients is especially complicated. Immunosuppressant doses should be high enough to maintain the intact function of the transplanted organ, and low enough to prevent the side effects and progression of the viral disease of COVID-19 [7]. Another consideration in SOT recipients is the bacterial and fungal co-infections with SARS-COV-2. In Wuhan, a study reported 15% prevalence of secondary bacterial infections among the hospitalized patients; meanwhile, the non-survivor group had a considerably higher prevalence of bacterial co-infections, compared to the survivors (50% vs. 1%; respectively). In the study, immune dysregulation and prolonged hospitalization were among the risk factors associated with nosocomial infections in COVID-19 patients [8].

Considering the significant role of co-infections in management and prognosis of COVID-19 in SOT patients, this study was done on the SOT recipients, hospitalized due to COVID-19, in Shiraz Transplant center, Iran, as the main center of SOT in the Middle East. The prevalence, type, risk factors and prognosis of bacterial and fungal co-infections with SARS-COV-2 in SOT patients were assessed in the current study.

## Methods

### Study setting and patients

The study was designed as a retrospective observational analysis of all the SOT patients admitted in the COVID-19 ICU of Abu-Ali Sina Hospital of Shiraz University of Medical Sciences, Iran from March 2020 to October 2020. This study was approved by the ethics committee of Shiraz University of Medical Sciences (Ethical Code: IR.SUMS.REC.1399.398).

The 30-bedded COVID-19 ICU was founded since the beginning of the pandemy specializing for admission of SOT recipients. COVID-19 was diagnosed either by positive SARS-CoV-2 reverse transcription polymerase chain reaction (RT-PCR) or by clinical symptoms with typical radiologic findings. The patients with negative RT-PCR for SARS-CoV-2 were excluded from the study.

Daily visits of patients was done by a multidisciplinary team consisting of a hepatobiliary surgeon, an infectious disease specialist, a nephrologist, an internist and a clinical pharmacist focusing on antiviral therapy, corticosteroid administration and managing the complications of the diseases.

### Data collection

The Demographic and laboratory data, microbiological results, medical interventions, disease outcome, length of mechanical ventilation, ICU and hospitalization and complications of COVID-19 were recorded and accessed from the available electronic documents of the patients. The documented bacterial and fungal infections were re-evaluated by the infectious diseases specialist.

### Procedures and definitions

Nasopharyngeal and oral swabs were used to obtain samples from suspected COVID-19 patients. The samples were tested at a central laboratory using real-time RT-PCR according to the WHO criteria [9]. Based on the clinical and laboratory findings, microbiological cultures from either blood, urine, or sputum were taken. Chest X-ray or lung computed tomography (CT) were demanded for the patients with suspected pneumonia. Blood cultures were obtained by the standard procedure [10]. The automats used were the BacT/ALERT 3D-automated blood culture system (bioMérieux, Durham, NC, USA) and the BACTEC FX (BD Diagnostic Systems, Sparks, MD, USA) (FX) for rapid microbial detection. Results were interpreted according to the clinical and laboratory standards institute (CLSI) guideline [11]. An expert infectious disease specialist exploited the clinical guidelines of the centers for disease control (CDC) for diagnosis of bacterial and fungal infections [12].

Co-infection was defined by clinical signs and/or symptoms of bacterial or fungal infection alongside with detection of a pathogen by a diagnostic test.

The classification of infections was based on the CDC/NHSN surveillance classification of healthcare-associated infections and criteria for specific types of infections in the acute care setting [12]. Sepsis diagnosis is based on Surviving sepsis campaign [13].

For each episode of infection, the symptoms, duration, site of infection, the isolated pathogens, prognosis and complications were recorded. Multidrug‐resistant (MDR) and Extensive Drug Resistance (XDR) pathogens were defined in accordance with the Consensus Statement of the European Centre for Disease Prevention and Control and the American CDC evidence of infection [14].

### Statistical analysis

The statistical analyses were performed by statistical package for social sciences (SPSS Inc., Chicago, Illinois, USA) version 16.0. Descriptive statistics were presented as mean ± SD and proportions as appropriate. Factors affecting infections were assessed by using binary logistic regression model and multivariate analyses. The Chi-square test or Fisher's exact test were used to compare the categorical data. The statistical significance level was set at p < 0.05.

## Results

During the duration of the study 97 adult and pediatric SOT recipients were admitted in the COVID-19 ICU, 68% (66 patients) of whom had positive RT-PCR for SARS-CoV-2 and the rest were diagnosed by clinical and radiologic findings. Three of the patients aged less than 18 years old. The 31 patients with negative RT-PCR were excluded from the study. The patient population consisted of 32 liver and 34 kidney recipients. The mean ± SD age of the patients was 55.22 ± 9.91 years. The majority of the patients were male (59.1% of patients). The demographic, comorbidity and disease data of the patients are presented in Table 1.

**Table 1:** Demographic and clinical characteristics of solid organ recipients with positive RT-PCR for SARS-CoV-2 (N = 66)

Patient/disease characteristics	Total patients (n = 66)	Co-infected group (n = 14)	The non-co-infected group (n = 52)	P value
Mean age (year)	55.22 ± 9.91	59.1 ± 11.2	54.01 ± 8.89	0.61
*Sex*				
Male	39	11	28	0.75
Female	27	3	24	
*Transplanted organ*				
Liver	32	9	23	0.89
Kidney	34	5	29	0.55
*Comorbid disease*				
Diabetes	22	11	11	p > 0.05
Hypertension	31	7	24	
Ischemic heart disease	37	11	20	
Chronic obstructive Pulmonary disease/asthma	1	1	0	
*Time after transplant (months)*				
< 6 months	38	11	27	0.11
> 6 months	28	3	25	
*Oxygen therapy*				
Nasal cannula	41	9	32	0.06
Mechanical ventilation	18	5	13	
None	7	0	7	
WBC (× 10^3^/L)	12.21 ± 4.31	14.69 ± 7.01	11.5 ± 5.91	0.49
CRP *(*mg/L)	75 ± 21	122 ± 31	48 ± 22	0.04
LDH (U/L)	241 ± 89	256 ± 91	213 ± 101	0.75
Length of hospitalization (day)	–	17.01 ± 2.11	12.24 ± 3.71	0.22
Length of mechanical ventilation (day)	–	11 ± 4	8 ± 4	0.17
Mortality	6	5	1	0.01
Rejection	1	0	1	0.91
Length of ICU stay	–	19.01 ± 10.00	8.90 ± 4.02	p < .001

*CRP* C-reactive protein, *ICU* intensive care unit, *LDH* Lactate dehydrogenase, *WBC* white blood cell

A total number of 14 patients (21.2%) had an overall 22 episodes of bacterial or fungal co-infections with SARS-CoV-2. Type of the transplanted organ (kidney vs liver), comorbid diseases, need for oxygen therapy and time passed from the transplantation were not significantly associated with increased risk of co-infections. The co-infected group had a significantly higher C-reactive protein (CRP) compared to the non-infected ones (122 ± 31 mg/L vs 48 ± 22 mg/L; respectively p = 0.04), whereas the white blood cell (WBC) count was not associated with bacterial and fungal co-infection (14.69 ± 7.01 10^3^/μL in the co-infected group vs. 11.5 ± 5.91 10^3^/μL in the non-co-infected group, p = 0.49). Among the 14 patients with co-infections, 5 individuals eventually passed away (35.7%), while 1 out of the 52 patients in the non-co-infected group died during the hospital course (1.9%). The presence of bacterial and fungal co-infections with SARS-CoV-2 was significantly associated with mortality (p = 0.01). A significantly longer ICU stay was recorded in the co-infected group compared to the non-co-infected patients (19 ± 10 days vs. 8 ± 4 days; respectively, p < 0.0001).

Urine and sputum constituted the majority sites of pathogen isolation (45.4% and 36.3% of infectious sources; respectively). *Enterococcus* spp, *Escherichia coli* and non*-albicans Candida* species were the most common isolated pathogens from the patients (30%, 23.3% and 13.3%; respectively). Among the infections, 9 episodes were co-infections with 2 pathogens (39.1%). A high prevalence of antibiotic-resistant bacterial strains was detected in the study. Among the 9 isolated *Enterococci*, 5 were of the vancomycin-resistant (VRE) type (55.5%). Both the 2 isolated *Staphylococci* spp were Methicillin-resistant *Staphylococcus aureus* (MRSA) and 6 of the 9 isolated *Enterobacteriaceae* species were Carbapenem-resistant *Enterobacteriaceae* (CRE) (66.66%). The infectious sources and isolated pathogens are demonstrated in Table 2.

**Table 2:** Isolated pathogens and site of infections among solid organ recipients affected with COVID-19

Infection characteristics	Number/percent
Site of pathogen isolation (n = 22)	
Urine	10 (45.45%)
Sputum	8 (36.36%)
Abdominal/pleural fluid	2 (9.09%)
Wound	1 (4.54%)
Blood	1 (4.54%)
Isolated microorganisms (n = 23)	
*Enterococcus* spp*.*	9 (30%)
*Escherichia coli*	7 (23.3%)
*Candida non-albicans*	4 (13.3%)
*Candida albicans*	1 (3.3%)
* Klebsiella* spp*.*	3 (10%)
*Pseudomonas* spp*.*	2 (6.6%)
*Acinetobacter* spp*.*	2 (6.6%)
MRSA	2 (6.6%)

Univariate regression analysis showed that diabetes mellitus, high dose of corticosteroids, history of hospitalization prior to COVID-19 and the length of hospital stay were the risk factors associated with bacterial or fungal co-infections with SARS-CoV-2. The multivariate analysis showed that among the mentioned risk factors, high dose of corticosteroids and prior hospitalization were the associated risk factors of bacterial and fungal co-infections with COVID-19. Table 3. Shows the results of the regression analysis.

**Table 3:** Univariate and multivariate analysis for the risk of bacterial and fungal co-infections among solid organ recipients affected with COVID-19

Variables	OR (95%CI) (P value) Univariate	OR (95%CI) (P value) Multivariate
Sex	1.21 (0.98–1. 27) (.78)	–
Age	0.91 (.77–2.29) (.92)	–
Kidney transplantation (N = 34)	1.01 (.23–5.42) (0.22)	–
Liver transplantation (N = 32)	2.29 (.11–4.98) (0.33)	–
Tacrolimus level	0.21 (− .01.1–2.92) (.91)	–
High dose of corticosteroids (N = 37)	5.65 (2.44–7.91) (0.03)	3.91 (2.02–6.90) (0.02)
Diabetes mellitus (N = 22)	4.94 (3.41–6.98) (< 0.001)	2.11 (0.96–3.41) (0.79)
Hypertension (N = 31)	21.20 (0.9–23.94) (0.76)	–
Length of hospitalization	1.10 (1.03–1.56) (0.03)	2.19 (0.81–6.71) (0.79)
Prior hospitalization before COVID-19	2.94 (2.77–6.79) (0.02)	3.66 (2.89–7.99) (< 0.001)

## Discussion

Immunocompromised patients are among the high risk groups to be infected with the novel SARS-CoV-2 virus [15]. The immunocompromised, more frequently tend to be admitted in the COVID-19 ICUs, more requently end up with mechanical ventilation and have higher mortality rates [16]. In a New York City study of 90 SOT patients, the mortality rate of COVID-19 was reported to be 18%, significantly higher than the normal population [17]. Efforts to maintain the intact function of the transplanted organ and concurrent reduction of the immune suppression dose to prevent severe viral disease, makes the management of COVID-19 in SOT patients remarkably challenging.

Due to immunosuppressive therapy, infections are already a frequent hazard in management of SOT patients. Bacteria are the most common pathogens causing infections after SOT, particularly in the early post-transplantation period [18, 19]. Multiple previous hospitalizations, invasive interventions and prior antibiotic use makes the SOT patients a susceptible population to colonize nosocomial and antibiotic resistant pathogens including MRSA, VRE and resistant gram negative bacilli [20]. On the other hand, community acquired infections may cause severe diseases in SOT patients, for instance bacterial or fungal superinfections exacerbate the common viral respiratory diseases in this vulnerable patient population [21].

Co-infections in general COVID-19 patients are not a frequent event, thus routine administration of antimicrobial agents is not recommended [22]. Bacterial and fungal infections are reported to co-exist with SARS-CoV-2 to be 8% among 806 patients integrated in a meta-analysis of the available worldwide studies [23]. In another meta-analysis of 3834 COVID-19 patients, the prevalence of co-infections was calculated to be 7%, in accordance with the previous study. In the study, *Mycoplasma pneumonia*, *Pseudomonas aeruginosa* and *Haemophilus influenzae* were the most common organisms to cause secondary bacterial infections [24].The superinfections were reported to occur as high as 44% in critically ill ICU patients [25]. Up to this date, few comprehensive studies of co-infections in COVID-19 SOT patients have been conducted. In this study we collected the clinical data of the 66 hospitalized SOT patients diagnosed with COVID-19 by positive SARS-CoV-2 RT-PCR. The prevalence of bacterial or fungal infections was 21.2% among our patient population which was as predicted, higher than non-SOT COVID-19 patients. In a French report of COVID-19 course in kidney transplant recipients, 23.5% of the patients had a secondary bacterial infection [26]. Patients with superinfections are at a higher risk of requiring ICU care and mechanical ventilation [27]. The incidence of bacterial co-infections in newly admitted COVID-19 patients is relatively low [28]. However, according to our study results, as ICU stay prolongs, the risk for co-infections increases. Furthermore, the need for mechanical ventilation increases the risk to acquire hospital-associated secondary pneumonia [29]. In the current analysis, prior hospitalization and high dose of corticosteroids were associated with increased risk of co-infection in SOT patients. In a previous study, older age was stated to be associated with more prevalent co-infections [30]. Nori et, al. have reported that 100% of the patients with MDR co-infections had received prior antibiotics [31]. Antimicrobial-resistant pathogens were frequent among our positive cultures, warning for an attentive antibiotic stewardship and avoiding empiric antibiotic prescription. Due to prior hospitalizations as a consequence of transplantation complications and receiving broad-spectrum antimicrobial agents, the high prevalence of MDR pathogens in SOT patients is a logical assumption [32, 33]. Of the MDR pathogens, VRE is of special concern as VRE infections contribute to various complications and twofold increased mortality of the liver transplantation patients [34]. The prevalence of VRE is differently reported worldwide and has high prevalence in some areas [32]. In our study, the most common isolated bacteria in COVID-19 SOT patients were VRE species. The majority of VRE infections occur in pre-colonized patients [35]. In a previous evaluation of pre-transplant patients in our center 9% were colonized with VRE [36]. Furthermore, the majority of the isolated *Candida* species were of the non-*albicans* type (80%). The emergence of non-*albicans Candida* species is a global phenomenon, as the use of azole antifungals is being frequently applied [37]. In the field of SOT, azoles are regularly prescribed as an anti-fungal prophylaxis. Therefore, an increasing trend in infections with non-*albicans Candida* rather than *Candida alibicans* is being observed [35].

Altogether, the most common type of secondary infection in COVID-19 patients is pneumonia, with blood stream and urinary tract infections standing at the next positions [27]. In the current study, urinary tract and sputum were the most common sites of pathogen isolation in the SOT patients, similar to the general COVID-19 patients. Ventilator-associated pneumonia was the most common co-infection in a surveillance of 52 SOT patients by Roberts et al. [38]. Superinfections should carefully be paid attention to, as they can be the terminal event resulting in death [39]. Secondary bacterial infections were detected in 50% of the COVID-19 patients who had died, according to Zhou et al. [8]. Martins-Filho et al., have reported a 2.5 fold mortality rate of COVID-19 in presence of co-infections [40]. In our study, the existence of bacterial and fungal co-infections contributed to a significantly high mortality rate (35.7%), while a small proportion of the non-co-infected group passed away (0.019%).

Differentiating COVID-19 progression and a secondary bacterial infection is challenging, as the inflammatory markers might be elevated in absence of co-infections. In our patient population, the co-infected group had a dramatically higher mean CRP level. CRP is usually elevated in the COVID-19 patients as a result of ongoing inflammation. In an extensive Chinese review, 60.7% of the patients had high blood CRP level [41]. Furthermore, a higher CRP level is associated with a more severe COVID-19 pneumonia and is an early predictive index of disease severity [42, 43]. Procalcitonin (PCT), the precursor of the thyroid hormone calcitonin, is produced by extra-thyroidal tissues during inflammatory response to bacterial components [44]. In non-bacterial inflammations, PCT is either in normal range or slightly elevated, which makes it a more desirable biomarker rather than CRP to predict bacterial co-infection [45]. One limitation of our study was, due to its retrospective nature, the scarce measurement and limited data of PCT levels in patients. Another limitation of our study was the limited number of patients, since it was a single center experience. Further studies of COVID-19 and co-infections in SOT patients are encouraged.

## Conclusions

SOT patients are a vulnerable population to be infected with SARS-CoV-2. Prior hospitalizations and immunosuppressive therapy makes them a susceptible group to contact bacterial or fungal infections during COVID-19 course. Although bacterial and fungal co-infection prevalence is higher in SOT patients, precise antibiotic stewardship is still recommended to prevent the emergence of multi-drug resistant organisms.

## Data Availability

Not applicable.

## Abbreviations

MRSA: Methicillin-resistant *Staphylococcus aureus*
PCT: Procalcitonin
RT-PCR: Reverse transcription polymerase chain reaction
SOT: Solid organ transplant
*VRE*: Vancomycin-resistant *Enterococcus*
